# Induction of IL-10-Producing CD1d^high^CD5^+^ Regulatory B Cells following *Babesia microti*-Infection

**DOI:** 10.1371/journal.pone.0046553

**Published:** 2012-10-05

**Authors:** Young-Il Jeong, Sung-Hee Hong, Shin-Hyeong Cho, Won-Ja Lee, Sang-Eun Lee

**Affiliations:** Division of Malaria & Parasitic Disease, Korea National Institute of Health, Cheongwon-gun, Chungbuk, Korea; National Jewish Health and University of Colorado School of Medicine, United States of America

## Abstract

**Background:**

Understanding the induction of immune regulatory cells upon helminth infection is important for understanding the control of autoimmunity and allergic inflammation in helminth infection. *Babesia microti*, an intraerythrocytic protozoan of the genus *Babesia*, is a major cause of the emerging human disease babesiosis, an asymptomatic malaria-like disease. We examined the influence of acute *B. microti* infection on the development of regulatory B cells together with regulatory T cells.

**Principal Findings:**

Our data demonstrate that B cells stimulated *in vitro* with *B. microti* produce interleukin (IL)-10. This cytokine is also secreted by B cells isolated from *B. microti*-infected mice in response to lipopolysaccharide stimulation. In addition, high levels of IL-10 were detected in the serum of mice after infection with *B. microti*. The frequency of IL-10-producing CD1d^high^CD5^+^ regulatory B cells (Bregs) and CD4^+^CD25^+^FoxP3^+^ T cells increased during the course of *B. microti* infection. Furthermore, adoptive transfer of IL-10-producing B cells induced by *B. microti* infection led to increased susceptibility of recipient mice to infection with *B. microti*. In contrast, experiments with B cell-deficient (µMT) mice demonstrated that lack of B cells enhances susceptibility to *B. microti* infection.

**Conclusions:**

This study is the first demonstration of the expansion of Bregs following infection by an intraerythrocytic protozoan parasite. These data suggest that *B. microti* infection in mice provides an excellent model for studying Breg-mediated immune responses and begins to elucidate the mechanism by which helminth infection regulates autoimmunity and allergic inflammation.

## Introduction

Recent clinical and experimental animal studies of allergic inflammation have demonstrated that helminths and their antigens can induce the suppression of hyperinflammatory responses [1], [2], [3]. This inverse relationship between the allergic immune response and helminth infection has been described as the ‘Hygiene Hypothesis’, which postulates that a steady decline in exposure to viral, bacterial, and parasitic infection leads to an increase in allergic disorders [4]. Because bacterial or viral infection induces the Th1 response, it is likely that infection by these pathogens leads to a concomitant suppression of the Th2 response. Because helminthic infections have been established to induce the Th2 response, their protective mechanisms in allergic disorders characterized by Th2-mediated inflammation are not easily explained. For this reason, much research has been focused on understanding how helminthic infection controls autoimmunity and allergic inflammation.

CD4^+^CD25^+^FoxP3^+^ regulatory T cells (Tregs) have been established to modulate autoimmunity and the allergic response. Previous studies have demonstrated the involvement of Tregs in mechanisms of helminthic infection that lead to protection against allergic disease. In fact, helminthes and their antigens have been shown to induce Tregs [2], [3], [5], [6]. For instance, *Schistosoma mansoni*-derived lyso-phosphatidylserine activates toll-like receptor 2 and induces Treg development by affecting dendritic cell maturation [6].

In addition to Tregs, several recent studies using experimental animal models of both allergic and autoimmune disease have identified a potential role for splenic B cells in suppressing the allergic or autoimmune response [7], [8], [9]. With their immunoglobulin secretion function, B cells are also able to produce regulatory cytokines such as interleukin (IL)-10, a key cytokine that inhibits cell-mediated immunity and inflammation while promoting humoral responses [10], [11].

This IL-10-producing regulatory B cell subset was first identified in B cell-deficient mice that failed to control experimental autoimmune encephalomyelitis (EAE) [12]. Yanabe, *et al.* identified a CD1d^high^CD5^+^CD19^+^ B cell population as a unique subset of potent regulatory B cells (Bregs) [9]. Breg-mediated suppression is important for maintaining peripheral tolerance and inhibiting harmful immune responses. This regulatory function appears to be mediated by the induction of Tregs via IL-10 secretion [7], [9]. Bregs have also been shown to be involved in suppressing allergic inflammation following parasitic infection. In fact, *Schistosoma mansoni* infection leads to the suppression of anaphylaxis and allergic airway inflammation via Breg induction [5], [13], [14], [15]. Therefore, investigating the induction of these immune regulatory cells in response to helminthic infection is important in furthering our understanding of the mechanism underlying the inverse relationship between allergic diseases and infection by this parasite.

*Babesia microti*, an intraerythrocytic protozoan of the genus *Babesia*, is a common parasite of wild rodents and a major cause of the emerging human disease babesiosis, an asymptomatic malaria-like disease [16], [17], [18]. Human infection by *B. microti* often occurs in diverse regions of North America and Europe, and is increasingly recognized as a health problem [19]. Babesiosis is often lethal in immunocompromised humans. Thus, the aim of this study was to investigate the influence of *B. microti* infection in promoting the expansion of immune regulatory cells, particularly IL-10-producing CD1d^high^CD5^+^ Bregs and the role of these cells in the susceptibility of mice to *B. microti* infection. We demonstrate that Bregs, Tregs, and IL-10 production induced by *B. microti* infection are required to facilitate the growth and survival of the parasite.

## Results

### Monitoring of *B. microti* infection and identification of babesiosis onset

**Figure 1:**
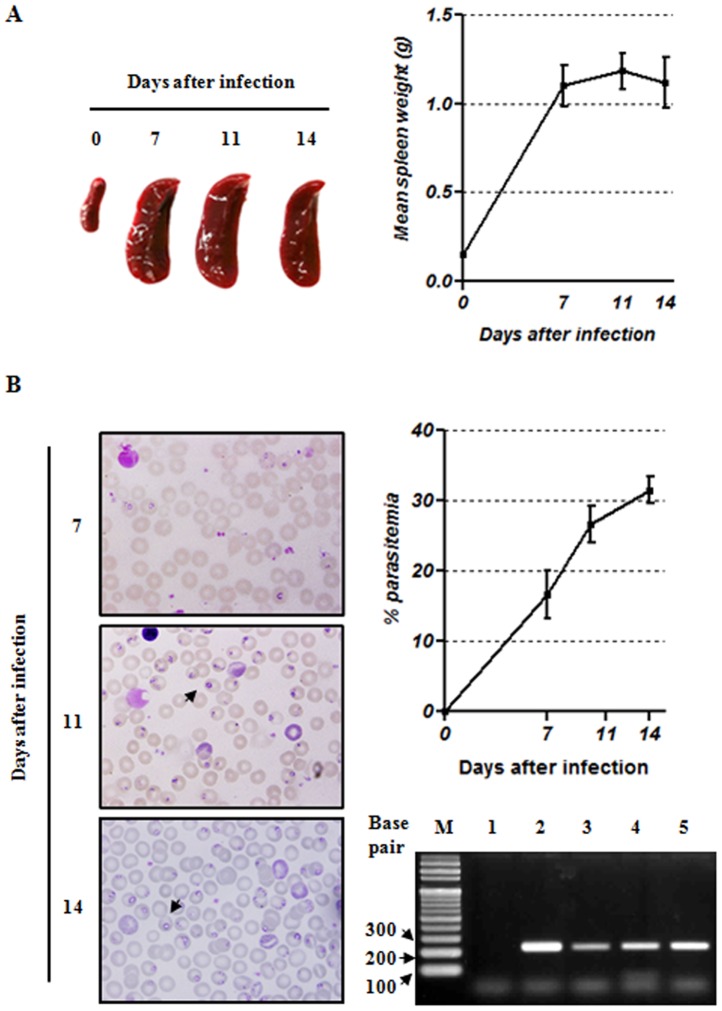
Enlargement of the spleen and parasitemia during the acute phase of *B. microti* infection. For the infection, 200 ml of an infected blood suspension (representing 20% parasitemia) was injected intraperitoneally. Control mice were injected with an equal total number of uninfected cells. (A) Enlargement and mean weight of spleens from *B. microti*-infected mice. (B) Identification of parasitized erythrocytes using Wright-Giemsa-stained blood smears (left panel) and the frequency of erythrocytes infected with *B. microti* versus the total number of red blood cells (right panel). Representative gel showing the PCR amplication of *B. microti*-specific 18S ribosomal ribonucleic acid genes (229 base pair segment) for the efficient detection of *B. microti* infection. Lane 1, negative genomic DNA control; Lane 2, positive control; Lane 3, day 7 after infection; Lane 4, day 11 after infection; Lane 5, day 14 after infection; Lane M, DNA ladder marker. All results represent at least five independent experiments with five mice in each group.

To establish a well-defined animal model for *B. microti* infection, we monitored babesiosis during the acute phase of infection. To confirm successful infection, spleens were weighed and parasitemia was calculated. At each time point, animals were sacrificed and their spleens were collected and weighed to within ±0.01 g. Enlargement of the spleen, which is caused by B cell proliferation, is a classic characteristic of babesiosis [16]. Consistent with a previous report [17], we observed that the mean splenic weight from infected mice increased as the infection progressed. From day 0 to day 7 post-infection, spleens from infected mice weighed up to six times more than those of uninfected control mice (Fig. 1A). Between day 7 and day 14 post-infection, the weight of infected spleens remained elevated. The course of infection, as revealed by examination of blood smears, is shown in Figure 1B. For the more sensitive and specific detection of babesial infection in peripheral blood, we also performed PCR assay. The highest level of parasitemia, which was represented by an average of 31.5% of infected erythrocytes, was observed at 14 days after infection. Therefore, these data suggest that acute infection started at day 7 and was fully established by day 14.

### *B. microti*-induced immune regulatory cells and anti-inflammatory cytokines facilitate rapid parasite growth and survival

**Figure 2:**
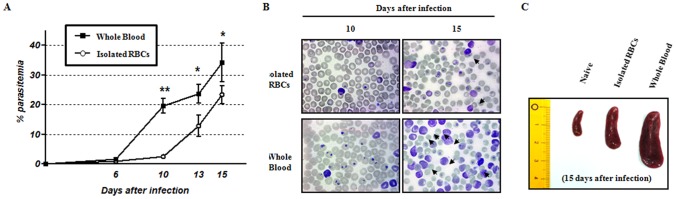
Comparison of susceptibility to *B. microti* infection between inoculation with purified RBCs alone versus whole blood. RBCs were separated from whole blood of *B. microti*-infected mice by Percoll gradient centrifugation, and then mice were inoculated with either whole blood or RBCs alone. (A) Development of parasitemia in mice infected with either purified RBCs alone or whole blood. The results are expressed as the mean percent parasitemia ± the standard deviation of five mice on the indicated day after infection. Asterisks indicate statistically significant differences between mice infected with whole blood versus isolated RBCs alone. *P<0.05 and **P<0.01 (B) Identification of damaged or dead RBCs using Wright-Giemsa-stained blood smears. Arrows indicate damaged or dead erythrocytes caused by *B. microti* infection. (C) Comparison of spleen enlargement. All results represent ≥two independent experiments with five mice in each group.

Whole blood obtained from initial *B. microti* infection contains various immune cells and soluble factors, such as cytokine and antibodies, stimulated by parasite infection. Because these cellular and humoral factors may be involved in controlling the rate of parasite growth, we determined the effect of Bregs, Tregs, and anti-inflammatory cytokines such as IL-10 on the susceptibility to *B. microti* infection. Pure parasitized RBCs were isolated from the whole blood of *B. microti*-infected mice by Percoll gradient centrifugation, and then mice were inoculated with either whole blood or purified RBCs alone. Interestingly, parasitemia in mice inoculated with isolated parasitized RBCs alone developed slower than that of mice infected with whole blood (Fig. 2A). These lower parasitemia levels persisted over 15 days after infection. Similarly, we observed that the number of damaged or dead RBCs was dramatically greater in animals inoculated with whole blood compared with those inoculated with parasitized RBCs alone (Fig. 2B). Moreover, inoculation with whole blood resulted in a larger spleen (Fig. 2C). These results indicate that immune regulatory cells and anti-inflammatory cytokines induced by *B. microti* infection are involved in facilitating rapid parasite growth and survival.

### Characterization of inflammatory cytokine production in response to *B. microti* infection

**Figure 3:**
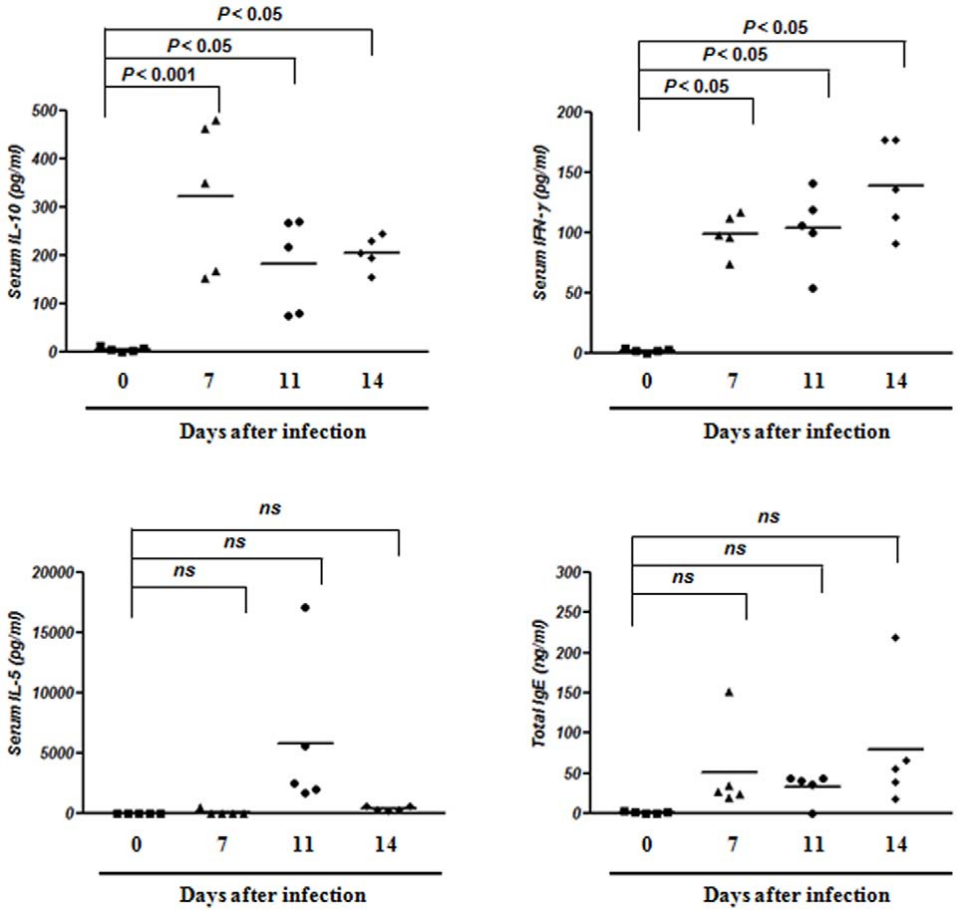
*B. microti* infection increases the levels of IL-10 and IFN-γ, but not IL-5 and total IgE, in serum. Blood samples of uninfected and infected mice were obtained by cardiac puncture. Then ELISA was performed to measure the levels of IFN-γ, IL-10, IL-5, and IgE in the serum. Each symbol represents one mouse; horizontal bar indicate the calculated me dian. IL-10 and IFN-γ production were increased significantly in infected mice compared to levels in uninfected mice. Data represent the mean ± the standard deviation of five mice per group. *P<0.05 and **P<0.001 indicates statistical significance compared to the uninfected control group. ns, not statistically significant. All results represent at least three independent experiments with five mice in each group.

Next, we examined whether cytokine responses were affected during acute infection with *B. microti*. To evaluate cytokine production in response to *B. microti* infection, serum was collected from infected mice at different days during the infection and analyzed by ELISA. The data in Figure 3 clearly show that, after infection with *B. microti*, a high level of IL-10 was detected in the serum throughout the experiment.

IFN-γ is essential for mediating host resistance and susceptibility to fatal infection with intracellular protozoan parasites such as *B. microti* and *Trypanosoma cruzi* [20]. IFN-γ production in infected mice was increased during the periods corresponding to the initiation and logarithmic phases of parasitemia; however, production of IL-4, IL-5, IL-13, and IL-12p70 remained very low, generally around the detection limit of the assay (Fig. 3 and data not shown). Total IgE production during the acute infection phase was similar at each infection time point assessed, with no significant increase observed compared to control mice. These results show that the production of Th2 cytokines and IgE was not affected by acute infection with *B. microti*.

### B cells stimulated *in vitro* with *B. microti* produce IL-10

**Figure 4:**
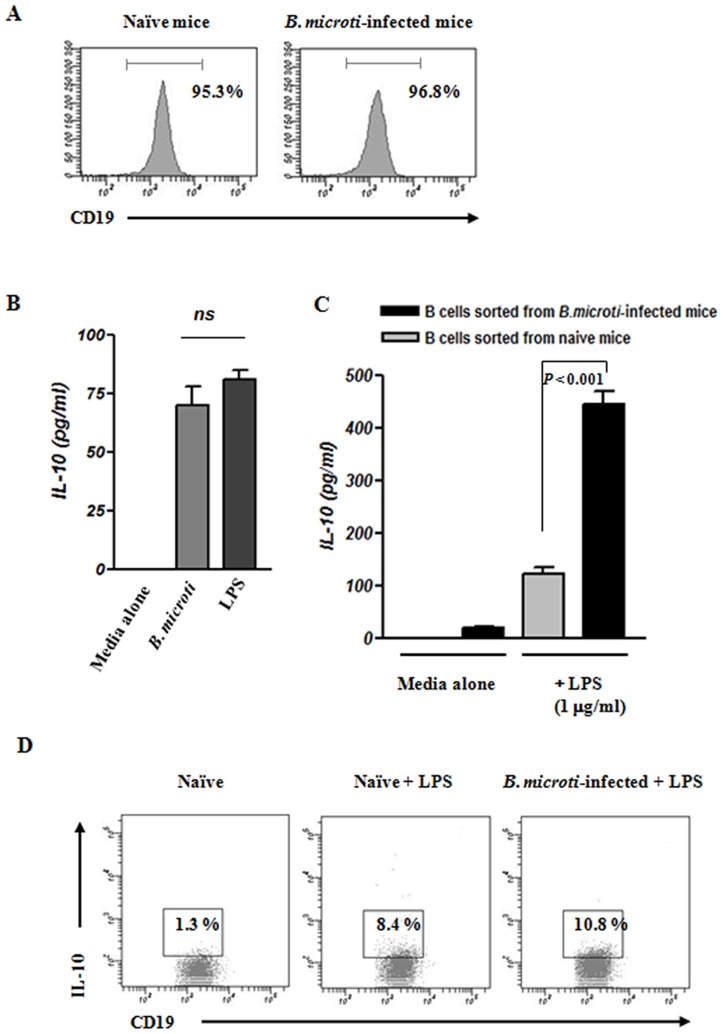
B cells secrete IL-10 in response to *B. microti* stimulation *in vitro*. Splenic B cells were purified from naïve or *B. microti*-infected mice by MACS as described in the *Materials and methods* (A) Representative histograms showing the purities of splenic CD19^+^ B cells isolated from either naïve (left) or *B. microti*-infected (right) mice. The purity of isolated B cells was >95% as assessed by flow cytometric analysis. (B) B cells sorted from naïve mice were stimulated with media alone, *B microti*, or LPS (1 µg/ml) for 24 h before the level of IL-10 in culture supernatants was determined by ELISA. (C) B cells isolated from either naïve mice or *B. microti*-infected mice were cultured with LPS for 5 h before the level of IL-10 in culture supernatants was measured by ELISA. (D) B cells were purified from naïve or *B. microti*-infected mice cultured with LPS for 5 h. The cells were then stained with anti-CD19 mAb, permeabilized, and stained intracellularly using anti-IL-10 mAb. Cytoplasmic IL-10 production was determined by flow cytometric analysis. Percentages indicate IL-10-producing cells among total CD19^+^ B cells. These *in vitro* experiments were repeated at least three times.

A recent study has demonstrated that IL-10 production from B cells is required for susceptibility to parasitic infection [11]. It also have been demonstrated that B cells purified from mice infected with parasite produced IL-10 in response to restimulation with parasite extract [11], [21], [22]. Thus, to determine whether B cells could produce IL-10 following stimulation with *B. microti in vitro*, B cells purified from naïve mice were purified (>95% purities, Fig. 4A). Similar to stimulation with lipopolysaccharide (LPS; 1 µg/ml), which was used as a control, B cells stimulated with *B. microti* secreted high levels of IL-10 *in vitro* (Fig. 4B). In addition, we assessed the effects of stimulating splenic B cells isolated from naïve or *B. microti*-infected mice with LPS. Our results demonstrate that LPS-stimulated B cells sorted from *B. microti*-infected mice produced significantly more IL-10 than control animals (3.6-fold; *P*<0.001; Fig. 4C). Flow cytometric analysis showed that intracellular IL-10 production was also higher in B cells sorted from *B. microti*-infected mice (Fig. 4D). These data indicate that B cells stimulated by *B. microti* infection play an important role in providing IL-10 for the development of susceptibility to parasite infection.

### *B. microti* infection induces expansion of IL-10-producing CD1d^high^CD5^+^ Bregs

**Figure 5:**
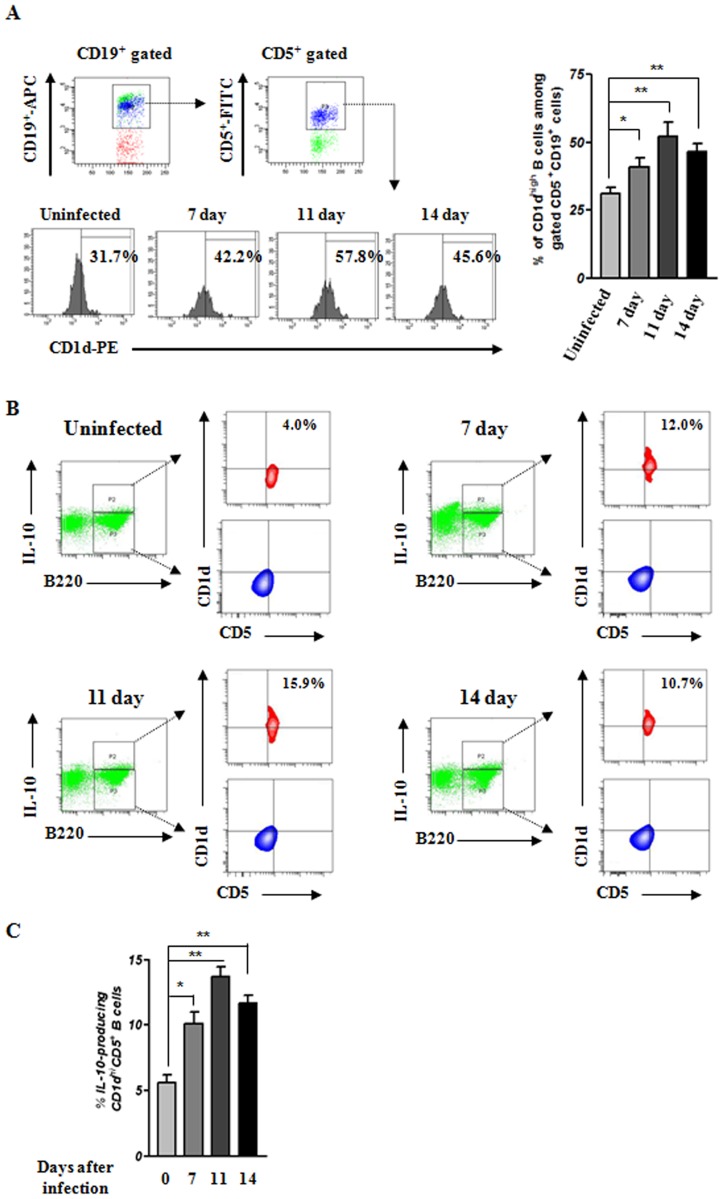
Identification of splenic IL-10-producing CD1d^high^CD5^+^ Breg population induced by *B. microti* infection. Splenocytes were isolated from uninfected mice and at days 7, 11, and 14 after *B. microti* infection. (A) Representative flow cytometric data shows the frequency of CD1d^high^ B cells among gated CD5^+^CD19^+^ B cells. Bar graph indicate the mean mean percentages ± the standard deviation of CD1d^high^ CD5^+^CD19^+^ B cells in one representative experimental group consisting of five mice. (B) To examine the capacity of *B. microti* infection to induce IL-10-producing CD1d^high^CD5^+^ Bregs, splenocytes isolated from infected or uninfected mice were stained with antibodies against CD19, B220, CD1d, CD5, and IL-10. The gating strategy used to identify IL-10-producing CD1d^high^CD5^+^ B cell is also depicted. (C) Bar graphs indicate the mean percentages ± the standard deviation of splenic CD1d^high^CD5^+^B220^+^CD19^+^ B cells that produced IL-10 in one representative experimental group consisting of five mice. *P<0.01 and **P<0.001 compared with the group of uninfected mice. Results presented were obtained by analyzing cells from individual mice and represent five independent experiments.

IL-10-producing B cells have recently been shown to function as Bregs and defined phenotypically as CD1d^high^CD5^+^CD19^+^ [5], [9]. To determine whether *B. microti* infection selectively induces Bregs, we analyzed cell surface marker expression on various splenic B cell subpopulations in uninfected and infected mice. Our data in Figure 5A depict the frequency of CD1d^high^CD5^+^CD19^+^ B cells as a percentage of CD1d^high^ B cells among gated CD19^+^CD5^+^ B cells. More specifically, on days 7, 11, and 14, the percentage of CD1d^high^CD5^+^CD19^+^ B cells was greater in infected mice than in uninfected mice (Fig. 5A), demonstrating that *B. microti* infection can affect the expansion of IL-10-producing Bregs.

As reported previously, B cells expressing high levels of CD1d and CD5 have been linked to the regulation of chronic intestinal inflammation, experimental arthritis, and T cell-mediated inflammation through IL-10 secretion [9], [23]. Thus, we sought to determine the frequency of IL-10-secreting CD1d^high^CD5^+^ B cells. To accomplish this, we analyzed the percentage of CD19^+^B220^+^CD1d^high^CD5^+^IL-10^+^ and CD19^+^B220^+^CD1d^high^CD5^+^IL-10^−^ splenocytes in uninfected and infected mice. Our data demonstrate that *B. microti* infection evokes expansion of IL-10-producing Bregs (Fig. 5B). Moreover, consistent with a previous study [9], the majority of IL-10-producing B cells exhibited a CD1d^high^CD5^+^ phenotype (Fig. 5B). This cell population also significantly increased on days 7, 11, and 14 after *B. microti* infection relative to uninfected mice (Fig. 5C). Collectively, these results suggest that *B. microti* infection induces the expansion of IL-10-producing CD11d^high^CD5^+^ B cell population in the spleen.

### Increase in CD4^+^CD25^+^FoxP3^+^ T-cell population by *B. microti* infection

**Figure 6:**
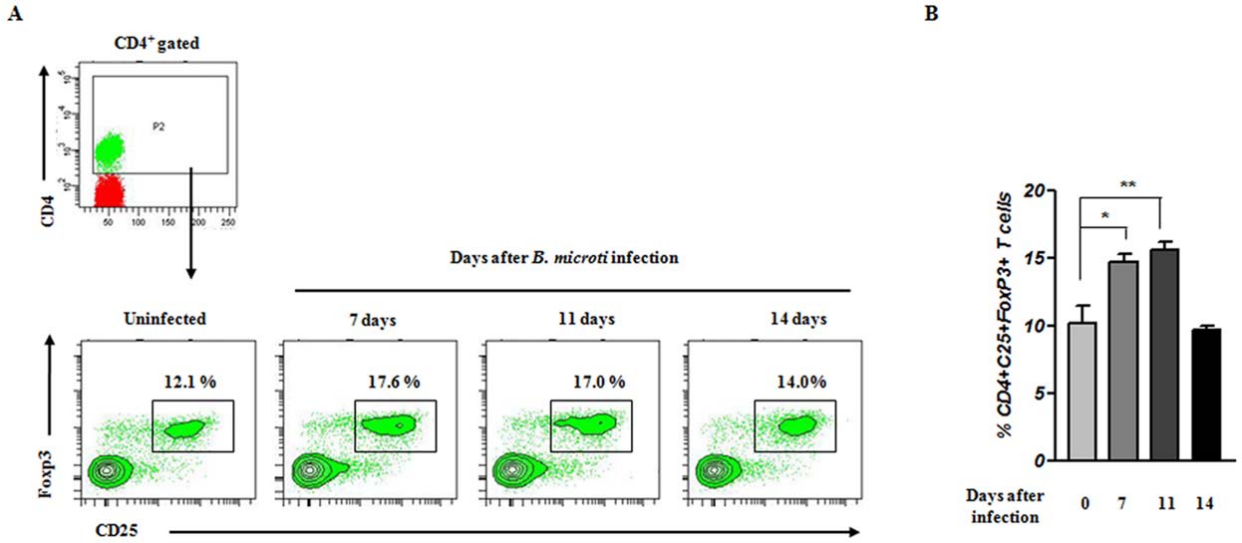
CD4^+^CD25^+^FoxP3^+^ T-cell frequencies increase during the course of *B. microti* infection. Splenocytes were stained with monoclonal antibodies against CD4 and CD25, permeablilized, stained intracellularly for FoxP3, and then analyzed by flow cytometry. (A) Representative data demonstrating the frequency of CD4^+^CD25^+^FoxP3^+^ T cells. The gating strategy used to identify CD4^+^ T cells is also depicted. (B) Bar graphs indicate the mean percentages ± standard deviation of splenic CD4^+^CD25^+^FoxP3^+^ T cells in one representative experimental group consisting of five mice. *P<0.01 and **P<0.001 compared with uninfected mice group. Results presented are from analysis of cells from individual mice and representative of five independent experiments.

Bregs have been widely implicated in promoting the expansion of Tregs and Treg-mediated suppression of allergic inflammation [5]. Previous studies have also demonstrated that following helminth infection, Bregs promote Treg expansion by secreting IL-10 [9], [23], [24]. Thus, we investigated whether the observed increase in Bregs following *B. microti* infection also leads to Treg induction. Flow cytometry was performed to examine the frequency of CD4^+^CD25^+^FoxP3^+^ Tregs in the spleen of *B. microti*-infected mice. Our results show that the frequency of splenic Tregs was increased at days 7 and 11 after *B. microti* infection compared to control mice (Fig. 6). Therefore, both Bregs and Tregs were increased during the acute phase of *B. microti* infection.

### Adoptive transfer of Bregs induced by *B. microti* infection increases the susceptibility to parasite infection

**Figure 7:**
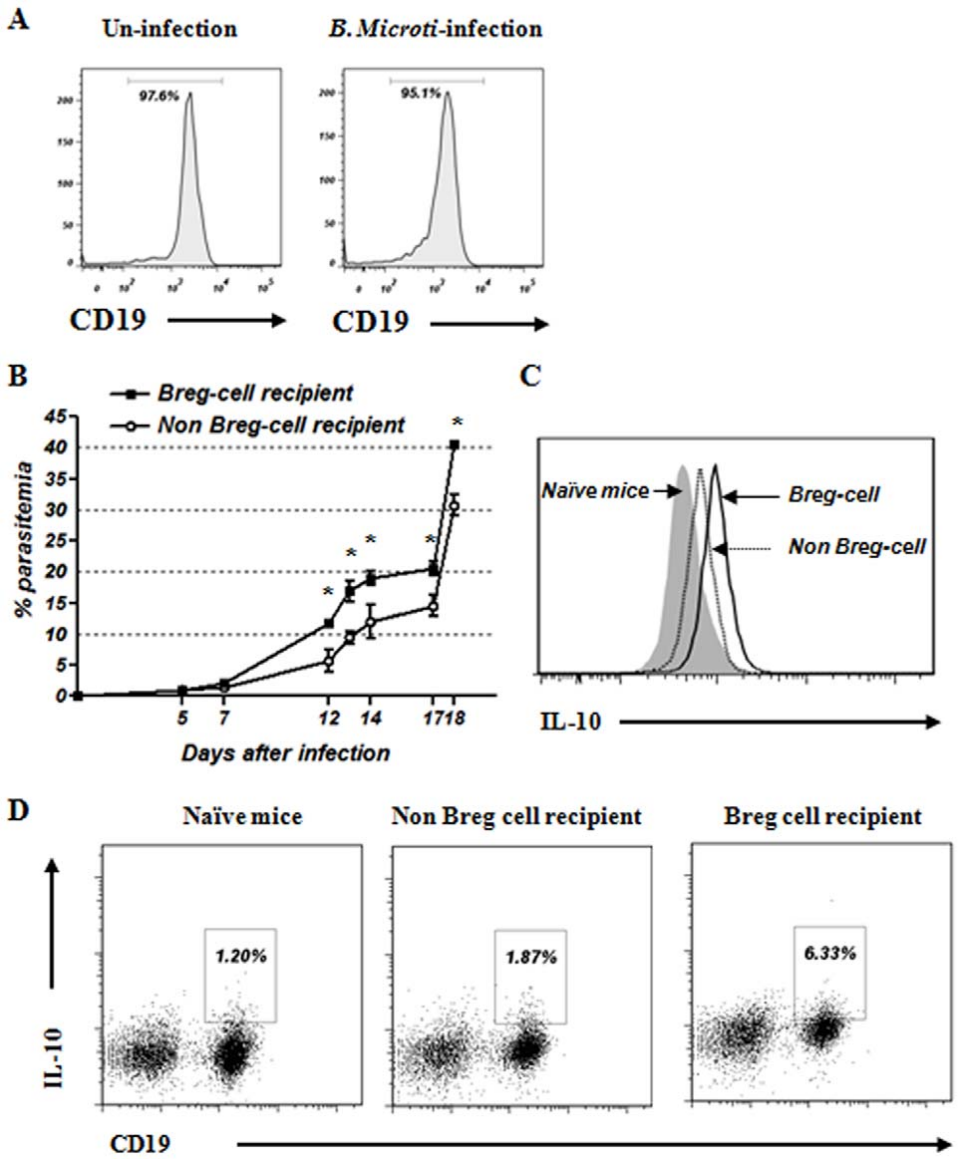
Adoptive transfer of IL-10-producing B cells isolated from *B. microti*-infected mice enhances susceptibility to *B. microti* infection. (A) IL-10-producing B cells were isolated from *B. microti*-infected mice. CD19^+^ B cells were isolated from naïve mice to be used as a control. On day 1 before *B. microti* infection, the sorted cells were transferred into recipient mice. (B) Development of parasitemia was monitored during the course of *B. microti* infection. The results are expressed as the mean percent parasitemia ± the standard deviation of five mice per group. Statistical significance between mean parasitemia in the Breg recipient and the Non Breg recipient groups is indicated. *, *P*<0.05. (C) Comparison of intracellular IL-10 production in CD19^+^ B cells of the Breg recipient and Non Breg recipient groups. The solid gray histogram represents uninfected naïve mice control. (D) Splenic IL-10-producing B cells from Breg recipient and Non Breg recipient groups. Values represent the percentage of IL-10-producing CD19^+^ B cells among total splenocytes. The data represent two independent infections.

To examine whether IL-10-producing Bregs induced by *B. microti* infection alter the susceptibility of *B. microti*-infected recipient mice, we performed adoptive transfer experiments. Splenic IL-10-producing B cells were isolated from *B. microti*-infected mice and transferred into recipient mice (termed Breg recipients) on days -1 relative to infection with *B. microti*. As a control, CD19^+^ B cells isolated from naïve mice were also transferred (termed Non Breg recipients) and inoculated with *B. microti* (Fig. 7A). While parasitemia was not enhanced in recipients given CD19^+^ B cells isolated from naïve mice, the transfer of IL-10-producing B cells isolated from infected mice induced greater parasitemia in recipient mice (Fig. 7B). Furthermore, intracellular IL-10 levels in splenic B cells from Breg recipients were increased relative to the control group (Fig. 7C). The IL-10-producing B cell population was also increased significantly in Breg recipients relative to the control group (Fig. 7D). These findings indicate that one important immunological function of Bregs induced by *B. microti* infection involves nurturing the parasitic environment by suppressing the host immune response.

### B cell depletion enhances the susceptibility to *B. microti* infection

**Figure 8:**
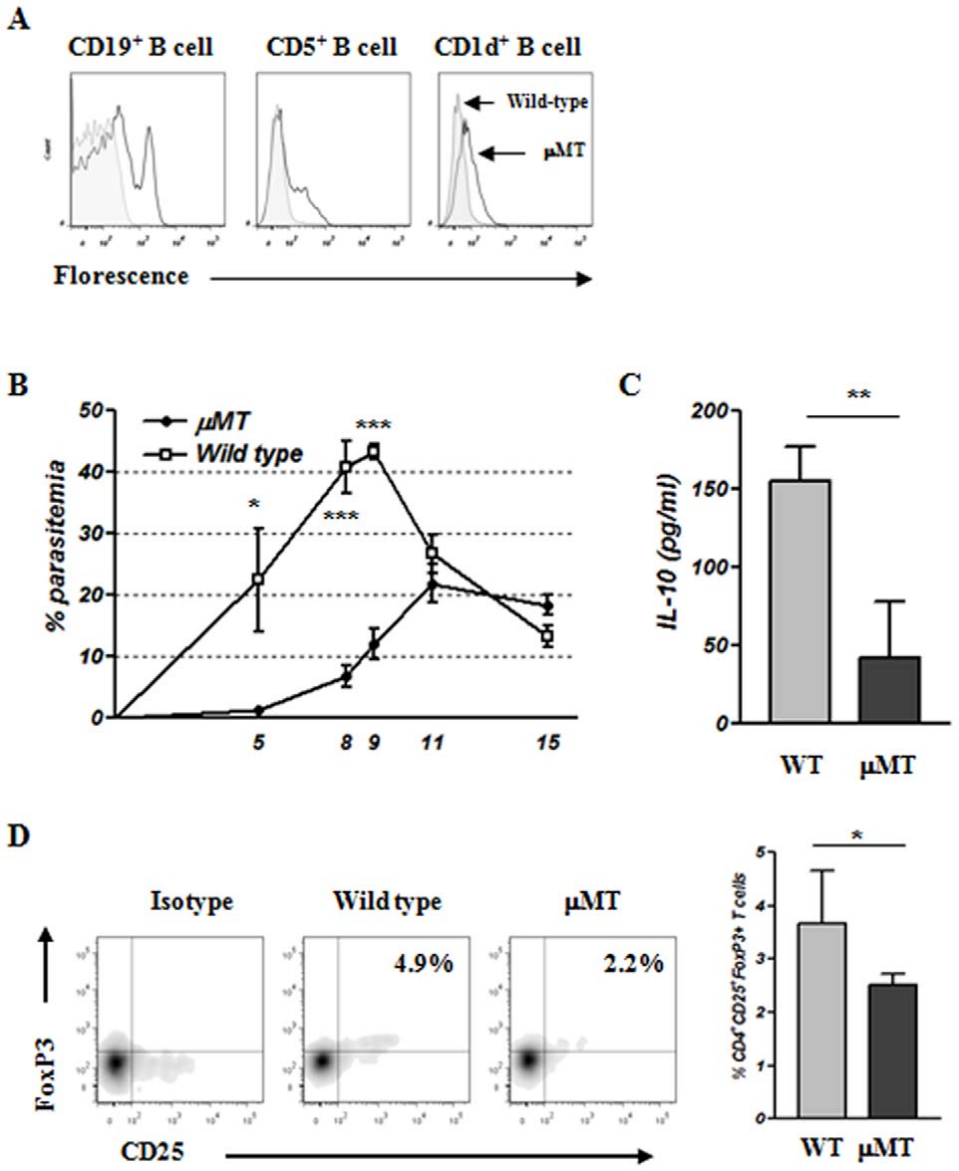
Lack of IL-10-producing B cells moderates the susceptibility to parasitic infection and induction of CD5^+^CD25^+^FoxP3^+^ T cells. (A) Identification of CD19, CD5, and CD1d expression in C57BL/6 µMT and C57BL/6 wild-type mice by flow cytometric analysis. The solid gray and white histograms represent wild-type and µMT mice respectively. (B) C57BL/6 µMT and C57BL/6 mice were inoculated with *B. microti*. Representative parasitemia profiles of both mouse strains throughout the 15 days of parasite infection are presented. (C) Blood was collected from wild type mice and µMT mice on final day of experiment. The production of IL-10 in mice after infection with *B. microti* was determined. (D) Comparison of CD5^+^CD25^+^FoxP3^+^ T cells in µMT and wild-type mice. CD5^+^CD25^+^FoxP3^+^ T cells in µMT and wild-type mice as determined by flow cytometric analysis. The frequency of CD5^+^CD25^+^FoxP3^+^ T cells was lower in µMT mice than in wild-type mice (right panel). Representative flow cytometric data from one experiment are shown. The results were comparable in two independent infections. The results are expressed as mean values ± standard deviation of five mice per group. Asterisks indicate statistically significant differences between wild-type versus µMT mice; *P<0.05, **P<0.01, and ***P<0.01.

Whether IL-10-producing B cells induced by *B. microti* infection are directly responsible for enhanced susceptibility to infection by this protozoan parasite was determined using the B cell-deficient mouse strain µMT, which lacks mature B cells. To examine the effect of Breg depletion on the outcome of *B. microti* infection, we first confirmed by flow cytometric analysis that mature B cells expressing CD19, CD5, or CD1d (i.e., Bregs) were not present in these mice (Fig. 8A). Interestingly, the development of parasitemia in µMT mice after *B. microti* infection was significantly slower and less severe than in wild-type mice (Fig. 8B). In addition, analysis of IL-10 production in serum showed that parasite-infected µMT mice produced lower levels of IL-10 than infected wild-type mice (Fig. 8C). Moreover, flow cytometric analysis of splenic Tregs on day 15 of infection revealed that, in the absence of Bregs, Treg development following parasite infection was reduced (Fig. 8D). These results indicate that IL-10-producing B cells induced by *B. microti* infection are involved in increasing the susceptibility to protozoan parasite infection and the induction of Tregs.

## Discussion

Our study investigated the influence of acute infection with *B. microti*, an intraerythrocytic protozoan parasite, on the development of Bregs and Tregs and the role of these immune regulatory cells in the susceptibility to parasite infection. Unlike trematode parasites such as *S. mansoni*, *B. microti* is a common protozoan parasite that invades and replicates within erythrocytes. The relationship between the development of immune cells with regulatory functions and trematode parasite infection has been studied extensively. However, immune regulation following infection by protozoan parasites remains poorly understood. To our knowledge, this is the first demonstration of the expansion of immune regulatory cells such as Bregs and Tregs following infection by an intraerythrocytic protozoan parasite.

Helminth parasites modulate multiple immune cell types and promote the generation of an anti-inflammatory environment that favors their survival, thus facilitating suppression of allergic-like inflammation [1], [5]. Our findings support the concept that various immune regulatory cells and soluble factors, such as IL-10, induced by *B. microti* infection play an important role in the growth and survival of this parasite. First, we demonstrated that removal of these cellular and humoral factors, which are initially *B. microti* infection, from whole blood leads to low susceptibility to infection with the parasite (Fig. 2).

IL-10 plays an important role in developing susceptibility to parasite infection by inhibiting cell-mediated immunity and inflammation [10], [11]. Although several different cell populations have been established as sources of IL-10 after parasite infection, secretion of this cytokine from B cells is important in the development of susceptibility to parasite infection [11]. Our data clearly demonstrate that following *B. microti* infection, B cells secrete IL-10, which influences the susceptibility of infection with *B. microti*. Indeed, high levels of IL-10 were detected in the serum of *B. microti*-infected mice throughout the experiment (Fig. 3). In addition, naïve B cells stimulated *in vitro* with *B. microti* produced high amounts of IL-10, similar B cells stimulated *in vitro* with LPS (Fig. 4B). Moreover, B cells sorted from *B. microti*-infected mice produced more IL-10 in response to LPS stimulation than B cells sorted from naïve mice (Fig. 4C and D). Finally, B cell-deficient µMT mice developed significantly lower levels of parasitemia after *B. microti* infection and exhibited lower levels of serum IL-10 compared to wild-type mice, suggesting that IL-10-producing B cells may play a role in susceptibility to infection with *B. microti* (Fig. 8B and C).

Recently, these IL-10-producing B cells, also known as Bregs, were identified in experimental models of autoimmune disease, cancer, and helminth infection. These cells are distinguishable by their cell surface markers and immunoregulatory function, which appears to be directly mediated by IL-10 production. Following parasite infection, IL-10 production by Bregs is important for maintaining peripheral tolerance by inhibiting B and T cell-mediated immune responses and inflammation necessary for efficient pathogen clearance. We show here that, during the acute phase of *B. microti* infection, the number of IL-10-producing CD1d^high^CD5^+^ Bregs is increased significantly (Fig. 5). We also demonstrate that these IL-10-producing Bregs play a critical role in the susceptibility to *B. microti* infection. Indeed, transfer of Bregs isolated from *B. microti*-infected mice increased parasitemia in recipient mice after *B. microti* infection (Fig. 7). Furthermore, studies using a B cell-deficient mouse strain, µMT, confirmed the important role of Bregs in susceptibility to *B. microti* infection (Fig. 8).

Amu and colleagues [5] suggested that Bregs may have a more systemic effect on Treg expansion in animal models of allergic disease. Other studies demonstrated that IL-10-producing Bregs function to downregulate autoimmunity and allergic disorders by inducing Tregs [5], [9], [24]. These findings can be extended to helminthic infection. In particular, adoptive transfer of *S. mansoni*-induced Bregs in allergic animal models resulted in IL-10-dependent induction of Tregs [5]. Also, IL-10 production was predominantly increased during parasite infection. This increased production explains the potent ability of Bregs to regulate T cell-mediated inflammatory responses [5], [9]. In accordance with these previous results, we demonstrate that *B. microti*-induced Bregs are associated with the induction of CD4^+^CD25^+^FoxP3^+^ Tregs. More specifically, the frequency of CD4^+^CD25^+^FoxP3^+^ Tregs increased concomitant with the development of IL-10-producing Bregs (Fig. 6). Most importantly, Treg induction by *B. microti* infection did not occur in the absence of IL-10-producing Bregs (Fig. 8D).

Finally, our results suggest that *B. microti* infection in mice provides an excellent model for studying Breg-mediated immune responses and begin to elucidate the mechanism by which helminth infection regulates autoimmunity and allergic inflammation. However, several questions remain to be addressed in future studies. First, why do both Breg and Treg populations decline during peak parasitemia? Second, would the Bregs and Tregs induced during the early phase of *B. microti* infection be lost upon long-term infection or after clearance of *B. microti*? In addition, whether Breg development induced by *B. microti* infection promotes Treg induction and suppression of the allergic inflammation response directly or indirectly remains to be determined. Based on the data presented in this study, we conclude that investigation into the immunological effects of *B. microti* infection may help in the development of new therapeutic strategies for treating allergic and autoimmune diseases.

## Materials and Methods

### Animals

Pathogen-free, female Balb/c and C57BL/6 mice, 5–6 weeks of age, were purchased from Orient Bio Inc (Korea). B cell-deficient µMT (B6.129S2-*Igh-6^tm1Cgn^*/J) mice were obtained from the Jackson Laboratory (Bar Harbor, ME, USA). These mice were housed in a specific pathogen-free facility in individually ventilated and filtered cages. All animals used in this study were maintained and handled in strict accordance with institutional guidelines. The experiments were approved by the Committee on the Ethics of Animal Experiments of the Korean Centers for Disease Control & Prevention (Permit number KCDC-004-11-2A).

### *B. microti* infection and parasitemia

*B. microti* (ATCC PRA-99, strain: Peabody mjr) was maintained and passaged in Balb/c mice before infection of experimental animals for this study. The percentage of parasitemia was determined by counting the number of parasitized erythrocytes versus the total number of red blood cells (RBCs): % parasitemia = (infected RBCs/RBCs)×100. A minimum of 500 RBCs were counted and an RBC infected with multiple parasites was counted as a single infected cell. A preliminary study was performed to determine the optimal infection dose and exposure scheme. For the infection, 200 µl of infected blood (20% parasitemia) suspension was injected into Balb/c mice intraperitoneally. Control mice were injected with an equal total number of uninfected cells. For injection with parasitized RBCs alone, we separated RBCs from whole blood of *B. microti*-infected mice by Percoll gradient centrifugation. Parasitemia after worm infection was identified by microscopic visualization of thin smears of tail blood stained with Wright-Giemsa stain (Merck, Darmstadt, Germany). To confirm *B. microti* infection, we performed the polymerase chain reaction (PCR) assay designed to amplify a 229-bp segment of *B. microti*-specific 18S ribosomal ribonucleic acid genes (5′-CTG CCT TAT CAT TAA TTT CGC ACG-3′ and 5′-ATG CCC CCA ACC GTT CCT ATT A-3′) as described previously with minor modifications [18]. Total DNA was extracted form 100 µl of whole blood using a commercially available kit (QIAamp DNA blood mini kit, Qiagen, Valencia, CA, USA) and used as the template for each PCR amplification. The cycling conditions were 95°C for 5 min followed by 30 cycles of 95°C for 45 s, 59°C for 45 s, and 72°C for 45 s, and then concluded with a final extension at 72°C for 5 min.

### Antibodies and flow cytometric analysis

For flow cytometric analysis, splenocytes were isolated using a 40-µm nylon cell strainer (BD Biosciences, San Jose, CA, USA) and then RBCs were lysed using a buffer containing 0.14 NH_4_Cl and 0.017 M Tris-base, pH 7.5. The cells were stained with the following monoclonal antibodies: fluorescein isothiocyanate (FITC)-conjugated anti-CD19 (clone 1D3), phycoerythrin (PE)-conjugated anti-CD1d (clone 1B1) (BD Biosciences); PE-Cy7-conjugated anti-CD5 (clone 53-7.3), peridinin chlorophyll-protein complex (PerCP)-conjugated anti-IL-10 (clone JES5-16E3), allophycocyanin (APC)-conjugated anti-CD45R(B220) (clone RA3-6B2) (eBioscience, San Diego, CA, USA). Intracellular staining for IL-10 was performed according to the manufacturer's protocol (eBioscience). Cells with the light-scatter properties of lymphocytes were analyzed by 3–5 color flow cytometry using the FACSCanto flow cytometer (Becton Dickinson, San Jose, CA, USA). To determine background staining, we used non-reactive isotype-matched control monoclonal antibodies (eBioscience) and gated to exclude ≥98% of non-reactive cells.

### Cytokine analysis

Changes in serum IL-10, interferon (IFN)-γ, IL-12p70, and IL-5 levels in mice infected by *B. microti* at various time points after infection were measured by enzyme-linked immunosorbent assay (ELISA) as previously described [3].

### B cell isolation and stimulation

A regulatory B cell isolation kit (Miltenyi Biotech Inc, Auburn, CA, USA) was used to purify IL-10-producing B cells according to the manufacturer's instructions. For cytokine production, 4×10^5^ purified CD19^+^ B cells were cultured either with parasitized erythrocytes or with LPS (*Escherichia* serotype 0111. B4, Sigma). Culture supernatant was collected to assess cytokine production and cells were harvested for flow cytometric analysis after 24 h.

### Adoptive transfer of IL-10-producing B cells

To adoptively transfer of IL-10-producing B cells, splenic IL-10-producing B cells were isolated from *B. microti*-infected mice and transferred into recipient mice (2.5×10^6^ B cells/mouse) intravenously on day -1 relative to infection with *B. microti*. As a control, CD19^+^ B cells isolated from naïve mice were also adoptively transferred into separate animals.

### Statistical analysis

Statistical analysis of any significant differences between the means of all variables was conducted using one-way analysis of variance (GraphPad Prism 3.0; GraphPad Software, CA. USA). Tukey's multiple-comparison test was used for pairwise comparison of data from multiple groups. All comparisons with a p-value<0.05 were considered statistically significant.
